# Machine learning prediction of suicidal ideation, planning, and attempt among Korean adults: A population-based study

**DOI:** 10.1016/j.ssmph.2022.101231

**Published:** 2022-09-14

**Authors:** Jeongyoon Lee, Tae-Young Pak

**Affiliations:** a Convergence Program for Social Innovation, Sungkyunkwan University, Seoul, South Korea; b Department of Consumer Science and Convergence Program for Social Innovation, Sungkyunkwan University, Seoul, South Korea

**Keywords:** Suicidal ideation, Suicide planning, Self-harm, Machine learning, Predictive modeling

## Abstract

**Background:**

Suicide remains the leading cause of premature death in South Korea. This study aims to develop machine learning algorithms for screening Korean adults at risk for suicidal ideation and suicide planning or attempt.

**Methods:**

Two sets of balanced data for Korean adults aged 19–64 years were drawn from the 2012–2019 waves of the Korea Welfare Panel Study using the random down-sampling method (*N* = 3292 for the prediction of suicidal ideation, *N* = 488 for the prediction of suicide planning or attempt). Demographic, socioeconomic, and psychosocial characteristics were used to predict suicidal ideation and suicide planning or attempt. Four machine-learning classifiers (logistic regression, random forest, support vector machine, and extreme gradient boosting) were tuned and cross-validated.

**Results:**

All four algorithms demonstrated satisfactory classification performance in predicting suicidal ideation (sensitivity 0.808–0.853, accuracy 0.843–0.863) and suicide planning or attempt (sensitivity 0.814–0.861, accuracy 0.864–0.884). Extreme gradient boosting was the best-performing algorithm for predicting both suicidal outcomes. The most important predictors were depressive symptoms, self-esteem, income, consumption, and life satisfaction. The algorithms trained with the top two predictors, depressive symptoms and self-esteem, showed comparable classification performance in predicting suicidal ideation (sensitivity 0.801–0.839, accuracy 0.841–0.846) and suicide planning or attempt (sensitivity 0.814–0.837, accuracy 0.874–0.884).

**Limitations:**

Suicidal ideation and behaviors may be under-reported due to social desirability bias. Causality is not established.

**Discussion:**

More than 80% of individuals at risk for suicidal ideation and suicide planning or attempt could be predicted by a number of mental and socioeconomic characteristics of respondents. This finding suggests the potential of developing a quick screening tool based on the known risk factors and applying it to primary care or community settings for early intervention.

## Highlights

• This study develops machine learning models to predict suicidal ideation and behaviors.
• Logistic regression, random forest, support vector machine, and extreme gradient boosting are used.
• The algorithms correctly identifyed 80–90% of suicidal cases.
• The algorithms with the top two predictors (depressive symptoms and self-esteem) could achieve comparable accuracy.
• Our findings can be used to design a quick screening tool for use in primary care or community settings.

## 1 Introduction

Suicide is a major public health concern in South Korea (Korea hereafter). In 2019, there were 13195 cases of suicide, or roughly one death by suicide every 38 min (Korean Statistical Information Service, 2020). A survey of more than 18000 Korean adults indicated that about 3.8% seriously considered suicide at some point in any given year and 0.5% planned or attempted suicide (Pak & Choung, 2020). The high suicide rate in Korea is often termed a “suicide epidemic,” highlighting a large fraction of the population at risk for suicide (Raschke et al., 2022). Previous studies have indicated that suicide completers rarely seek psychiatric treatment before they commit suicide, although they often visit primary care providers to address physical symptoms associated with poor mental health (Zalsman et al., 2016). This suggests the potential of a prospective screening tool for use in primary care or community settings to identify potential recipients of preventive interventions (Raue et al., 2014).

Suicide is a complex, multifaceted phenomenon in which a constellation of risk markers mediates its pathogenic mechanisms (De Berardis et al., 2018). Shneidman's (1993) theory of suicide identifies psychache (i.e., psychological and emotional pain at intolerable intensity) as the primary motivator of suicidal desire, highlighting the role of mental illness. Baumeister's (1990) escape theory posits that self-blame for difficult life events and negative self-perceptions interact to confer the desire for suicide. Empirical studies found elevated levels of mental health symptoms, including anhedonia, anxiety disorders, depression, and self-esteem deficits, among those who reported suicidal ideation or suicide attempts (Bhar et al., 2008; Ducasse et al., 2018; Fredriksen et al., 2017). Emerging evidence also pointed to subjective assessment of life conditions as possible mechanisms through which other risk markers may operate (Heisel & Flett, 2004; Suh et al., 2021). In the Korean context, various dimensions of economic insecurity were associated with suicidal ideation and behaviors (Kim & You, 2019; Raschke et al., 2022; Yoon et al., 2017). Researchers have found that no single factor is sufficient to explain suicide, but several interacting factors jointly contribute to suicide risk (Kuroki & Tilley, 2012). Recent studies have called for a multifocal approach to suicide prediction, which considers a full spectrum of risk markers validated by theories and empirical research (Choi et al., 2018; Kuroki & Tilley, 2012; Passos et al., 2016).

Machine learning (ML) has emerged as a promising analytic tool for integrating complex risk markers into clinical signatures of suicide. The idea behind ML is to learn the typical characteristics of a class (e.g., those with suicidal symptoms) from past data and apply this knowledge to unseen data to identify a sample with similar characteristics. Typically, estimating a reliable ML algorithm requires a large number of inputs that contain useful information for distinguishing one group of subjects from others, and iteratively tuning the algorithms to achieve higher predictive accuracy. Early attempts to predict suicide have used unstructured text data, such as social media posts (Cash et al., 2013; Jashinsky et al., 2014) and counseling transcripts (Pestian et al., 2010), to detect distinctive patterns in natural language related to suicide. While these approaches demonstrated methodological potential of ML, they could not be used in the primary care or community setting because they rely on unconventional big data that is not available at the time of screening. Subsequent research showed that ML algorithms based on survey or clinical data could achieve a satisfactory level of accuracy in classifying individuals who are likely to think about suicide (Hill et al., 2017; Ryu et al., 2018), attempt suicide (Bae et al., 2015; Oh et al., 2017; Passos et al., 2016), and complete suicide (Choi et al., 2018; Jiang et al., 2021).

This study extends the previous literature by providing an empirical foundation for the prediction model via ML modeling of population-based longitudinal data. Specifically, this study explores whether the predictive signature of suicide derived from easily accessible demographic, socioeconomic, and psychosocial variables in the population data can help distinguish suicide-prone individuals from their nonsuicidal peers. Using a representative community sample of Korean adults, we estimated a series of ML algorithms that link individual- and household-level predictors to suicidal ideation and suicide planning or attempt. Predictors were selected based on a *priori* knowledge and related theories. Our ML models integrate the information from multiple predictors to subsequently estimate an individual's probability or risk of being a suicide ideator or a suicide planner/attempter over the next 12 months.

The prediction models developed here may help identify priority targets for prevention and intervention efforts. Suicidal ideation is an important precursor of suicide planning or attempt, with 15.6% of ideators going on to make an attempt within 12 months (Borges et al., 2006) and 31.8% making an attempt at some point in their lifetime (Nock et al., 2008). Among those who attempt suicide, a significant portion re-attempt suicide and eventually die (Suominen et al., 2004). Clinical studies commonly find that a substantial percentage of suicide ideators experience psychiatric illness or poor psychosocial conditions as they progress to attempt (see O'Connor et al., 2013). This background represents an important opportunity to prospectively screen people at risk for suicide and intervene with appropriate prevention measures. The application of ML to large-scale population data may help develop an efficient screening system for the general population.

## 2 Methods

### 2.1 Data description

This study used the 2012–2019 waves of the Korea Welfare Panel Study (KoWePS), conducted by the Korea Institute for Health and Social Affairs and Seoul National University. The KoWePS is a longitudinal cohort study that annually follows a nationally representative sample of South Korean households. The first study was conducted in 2006, with 18856 participants from 7072 households. The initial sample was selected from 16 provincial districts in proportion to the population size of each district using stratified multistage cluster sampling. Interviews were conducted by trained interviewers at the participants’ homes via computer assisted personal interview. The interviewers were individuals aged 18 or higher with experiences or interests in social surveys. After each survey, the KoWePS randomly selected 10% of the recorded responses and conducted a post-interview quality check. If the survey was not conducted according to the guideline, an additional interview was conducted over the phone. The topics included in KoWePS were demographic background, economic characteristics, social service needs, health status, healthcare utilization patterns, and psychosocial well-being. All participants provided informed consent before participating in the survey. The details of the survey protocol and sampling design are available elsewhere (https://www.koweps.re.kr/).

The study sample was restricted to individuals aged 19–64 years. The age of 19 is the age at which a Korean citizen is legally recognized as an adult. Those older than 64 years were not included in this study as their health and socioeconomic characteristics might be different from those of younger cohorts in unobserved ways.^1^ The baseline data included 60568 observations from 11114 individuals with no missing data. Each observation comprised 57 features (including two measures of suicidality) that were considered for predictive modeling.

### 2.2 Suicidal ideation and suicide planning or attempt

A binary indicator of suicidal ideation was based on an affirmative response to the interview question, “*Have you seriously considered committing suicide in the past year?*”. A binary indicator of suicide planning or attempt was based on an affirmative response to the questions, “*Have you made a concrete plan to commit suicide in the past year?*” or “*Have you made an attempt to commit suicide in the past year?*”. This coding scheme leads to 1646 person-level observations of suicidal ideation and 244 person-level observations of suicide planning or attempt. The two binary measures were used to label each observation in the classification problem below.

### 2.3 Class imbalance problem

Learning from data with a severely imbalanced target variable poses empirical challenges for ML research (Libbrecht & Noble, 2015). It is a particularly salient issue in suicide prediction because the size of the no-suicide-risk group far exceeds the size of the at-risk group (Passos et al., 2016). Under this setting, comparing predicted probabilities for suicidal outcome to a default cutoff of 0.5 leads to high specificity but low sensitivity, making it difficult to assess the algorithm's classification performance. One way to circumvent this issue is to under-sample the majority class (i.e., those with no risk of suicide) so that the sample is balanced across target labels (Passos et al., 2016; Ryu et al., 2018).^2^ In this study, we created two sets of balanced data: one for predicting suicidal ideation and one for predicting suicide planning or attempt. Specifically, a total of 1646 observations were randomly drawn from the pooled sample of no suicidal ideation, yielding a balanced dataset of 3292 observations (data A). We also randomly selected 244 observations from the pooled sample of no suicide planning or attempt to create a balanced dataset of 488 observations (data B). The average sample characteristics are presented in Table 1 with a number of representative features.

**Table 1:** Descriptive statistics.

	Full sample		Suicidal ideation		Suicide planning or attempt	
	(*N* = 60568)		(*N* = 3292)		(*N* = 488)	
	Mean	SD	Mean	SD	Mean	SD
Age (19–64)	43.9	12.2	45.7	12.2	45.9	12.4
Female (0,1)	0.54		0.57		0.60	
Education background (0,1)	0.45		0.34		0.31	
Marital status (0,1)	0.67		0.58		0.57	
No. of household members	3.28	1.21	3.01	1.29	2.98	1.26
Employment status (0,1)	0.71		0.62		0.56	
Region of residence (0,1)	0.40		0.41		0.43	
Religion (0,1)	0.46		0.45		0.46	
Household income (in 2019 KRW)	5962.5	5900.4	4803.3	3776.4	4598.4	3603.6
Household consumption (in 2019 KRW)	423.3	249.5	360.2	238.0	344.3	230.2
Household net worth (in 2019 KRW)	13625.9	36446.7	10024.8	31557.9	9378.9	32144.1
Social welfare receipt (0,1)	0.06		0.15		0.19	
No. of outpatient visits	10.2	19.4	16.7	30.0	18.6	32.6
Poor self-rated health (0,1)	0.25		0.40		0.45	
Disability (0,1)	0.06		0.12		0.14	
Any chronic disease (0,1)	0.37		0.49		0.55	
Smoking (0,1)	0.22		0.26		0.27	
Drinking (0,1)	0.59		0.55		0.51	
CESD score (0–33)	2.68	4.03	6.68	7.10	8.31	8.42
Self-esteem score (0–30)	21.3	3.81	18.8	5.15	17.8	5.86
Experience of physical abuse from spouse (0,1)	0.67		0.60		0.58	

*Notes*: N, number of observations; SD, standard deviation.

### 2.4 Predictor variables

A common practice in ML research is to select predictors based on expert knowledge, preferably published studies. Following Passos et al. (2016), we conducted a structured search of the PubMed database to identify published articles that reported the determinants of increased suicidal risk. In all, 55 predictors were selected according to the literature review and their availability in KoWePS (Supplementary Table S1). These include demographic, socioeconomic, health and well-being, and early life characteristics of participants and their households that are likely to be correlated with suicidal risk as predicted by underlying theories (Baumeister, 1990; Shneidman, 1993).

**Fig. 1:**
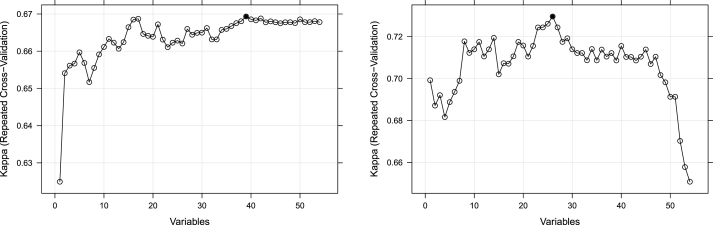
Recursive feature elimination results.

A recursive feature elimination (RFE) algorithm was used to identify a subset of predictors that ensured the highest classification performance. RFE is a feature selection method that recursively eliminates weak predictors to reduce dependencies and collinearity that may exist in the model. This study used logistic regression and 10-fold cross-validation with three repeats to evaluate the model's classification performance during the elimination process. We found that when the target label was suicidal ideation, the model trained with 39 predictors achieved the highest kappa value (Fig. 1). For the model that predicted suicide planning or attempt, 26 predictors led to the highest kappa. The selected predictors are shaded gray in Supplementary Table S2.

### 2.5 Machine learning algorithms

We employed four sets of predictive algorithms for comparison: logistic regression, support vector machine (SVM), random forest (RF), and extreme gradient boosting (XGBoost). Logistic regression assumes a linear relationship between the logit of the outcome and predictor variables, and has been widely used in ML research on suicide predictions (Jung et al., 2019; Kessler et al., 2017; van Mens et al., 2020; Walsh et al., 2017). The SVM finds a linear hyperplane that maximizes the separation or margin between two classes in a higher-dimensional feature space (Choi et al., 2018). It makes a nonlinear mapping of the original data into a higher dimension to form a decision surface suitable for classification. RF is a tree-based algorithm that ensembles a number of decision trees based on bootstrapped samples and aggregates votes (predicted class) from each tree (Breiman, 2001). It improves upon the classification tree by considering a random subspace of predictors when building a tree and by creating a diverse set of trees that contribute to classification performance. XGBoost is a scalable tree-boosting algorithm proposed by Chen and Guestrin (2016). The algorithm derives from the idea of boosting, which combines the prediction results of the “weak” learners with those of the “strong” learners through cumulative training instances. It has been shown to have desirable properties, including regularization, handling of missing values, flexible evaluation criteria, optimized computation processes, and high classification performance. Logistic regression has the advantage that it is fully interpretable and efficient for training. Other algorithms considered here have been shown to provide a more accurate classification than logistic regression when the data are linearly inseparable. We trained and tuned these four ML algorithms independently and compared their classification performances. All analyses were performed using R/RStudio version 4.1.0 (Integrated Development Environment for R, Boston, MA) and caret package (Kuhn, 2008).

### 2.6 Algorithm development and validation

A unique challenge of ML is that the algorithm's generalizability to unseen data cannot be assessed until future data arrives. Techniques such as cross-validation help evaluate the algorithm's out-of-sample performance by setting aside a portion of data as “unseen” and using the remaining data for algorithm training. In this study, we followed the strategy of Xue et al. (2018) of using more recent survey waves for algorithm testing and earlier survey waves for algorithm training. The implicit assumption here is that recent data are more reflective of future data and therefore more suitable for evaluating the algorithm's predictive performance. Specifically, we set aside the last two waves (2018 and 2019 surveys) as the test set and used the older waves as the training set.

**Fig. 2:**
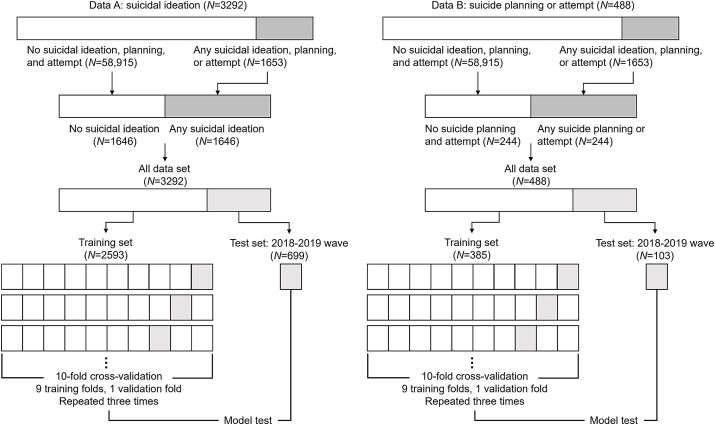
Data construction and model development.

For SVM, RF, and XGBoost, the hyperparameters were optimized using a grid search on 10 randomly selected training and validation sets. Grid search is a tuning technique that pursues the optimum values of hyperparameters through an exhaustive search. Testing each hyperparameter setup requires our data to be partitioned into training and validation sets. Here, we created 10 equally sized random folds of data, where each fold was used once as a validation set and the other nine folds were used for training. This evaluation process was repeated three times, and the classification performance of the algorithm was averaged over the repeats (10-fold cross-validation with three repeats). Each set of hyperparameters undergoes this evaluation process until we find the set with the highest classification accuracy (Fig. 2). SVM, RF, and XGBoost at the optimal setting, along with logistic regression, were used to predict suicidal outcomes in the test set. The classification performance of the final algorithm was assessed using the area under the curve (AUC), sensitivity, specificity, positive predictive value, negative predictive value, and accuracy. Our interpretation places greater emphasis on sensitivity because suicide prevention aims to minimize false negatives.

## 3 Results

Our data comprised of 3292 observations for predicting suicidal ideation and 488 observations for predicting suicide planning or attempt (Table 1). In a sample of 3292 observations, there were 57% female, 34% college graduates, and 58% married individuals. The mean age was 45.7, with a standard deviation of 12.2, and the mean number of family members was 3.01. The sample was predominantly employed (62%), non-smokers (74%), and non-disabled (88%). The sample for suicide planning or attempt exhibits similar characteristics, except that it includes greater share of welfare beneficiaries, disabled respondents, and those with poor self-rated health. This difference is consistent with our knowledge that suicide planning or attempt are more prevalent in disadvantaged populations.

Optimal hyperparameters were obtained for each ML algorithm using the grid search method (Table 2). We trained the SVM with three different kernels (linear, radial, and polynomial) and found that the linear kernel at the optimal cost achieved the highest accuracy with the validation set. The optimal setup for RF was determined through exhaustive evaluation of the algorithm at two possible split rules (Gini and extratrees) and varying degrees of minimal node size and the number of randomly selected predictors considered for a split. XGBoost was tuned over boosting iterations, maximum tree depth, shrinkage, minimum loss reduction, subsample ratio of columns, minimum sum of instance weight, and subsample percentage. The classification results below were generated by each algorithm using the optimal hyperparameters.

**Table 2:** Tuned hyperparameters.

Panel A: suicidal ideation (*N* = 3292)	
SVM	kernel = linear, cost = 1.5
RF	split rule = Gini, minimal node size = 2, number of randomly selected predictors = 6
XGBoost	number of boosting iterations = 120, max tree depth = 3, shrinkage = 0.04, minimum loss reduction = 2, subsample ratio of columns = 0.55, minimum sum of instance weight = 5, and subsample percentage = 1
Panel B: suicide planning or attempt (*N* = 488)	
SVM	kernel = linear, cost = 0.5
RF	split rule = Gini, minimal node size = 2, number of randomly selected predictors = 7
XGBoost	number of boosting iterations = 60, max tree depth = 5, shrinkage = 0.04, minimum loss reduction = 3, subsample ratio of columns = 0.5, minimum sum of instance weight = 6, and subsample percentage = 1

*Notes*: SVM, support vector machine; RF, random forest; XGBoost, extreme gradient boosting.

Table 3 presents the classification metrics of the four models predicting suicidal ideation (panel A) or suicide planning or attempt (panel B) in the test set. Panel A shows that the accuracy rate was highest for XGBoost (0.863), followed by RF (0.851), SVM (0.850), and logistic regression (0.843). The four algorithms showed a sensitivity of 0.808–0.853, meaning that approximately 80.8–85.3% of those who thought about committing suicide were correctly identified by the algorithms. Other metrics showed a specificity of 0.852–0.877, positive predictive value of 0.799–0.820, and negative predictive value of 0.867–0.895. The cross-validated AUC was highest for XGBoost (0.861) and lowest for logistic regression (0.837), both of which represent satisfactory classification performance. Overall, XGBoost appeared to provide more accurate classification results than the other algorithms when predicting suicidal ideation.

**Table 3:** Algorithm performance.

	Logistic	SVM	RF	XGBoost
Panel A: suicidal ideation (*N* = 3292)				
Area under the curve	0.837	0.844	0.851	0.861
Sensitivity	0.808	0.811	0.850	0.853
Specificity	0.867	0.877	0.852	0.869
Positive predictive value	0.808	0.820	0.799	0.819
Negative predictive value	0.867	0.870	0.891	0.895
Accuracy	0.843	0.850	0.851	0.863
Panel B: suicide planning or attempt (*N* = 488)				
Area under the curve	0.872	0.872	0.857	0.880
Sensitivity	0.861	0.861	0.814	0.861
Specificity	0.883	0.883	0.900	0.900
Positive predictive value	0.841	0.841	0.854	0.861
Negative predictive value	0.898	0.898	0.871	0.900
Accuracy	0.874	0.874	0.864	0.884

*Notes*: SVM, support vector machine; RF, random forest; XGBoost, extreme gradient boosting.

Panel B shows an accuracy of 0.864–0.884 and sensitivity of 0.814–0.861 across the four algorithms. The cross-validated AUCs were approximately 0.857–0.880, confirming the algorithm's generalizability over unseen data. XGBoost was still the best-performing algorithm, correctly identifying 86.1% of those who engaged in suicide planning or attempt. The other metrics showed similar results across the four algorithms. Overall, the algorithms predicting suicide planning or attempt generated slightly more accurate classifications than those predicting suicidal ideation.

Our approach to use more recent data as a test set could potentially lead to an overfitting problem as previous observations of the respondents were used to train the model. To evaluate this concern, we have re-estimated the algorithms in Table 3 using (a) the train and test data randomly drawn from the baseline sample without considering the timing of the survey (Table A1) and (b) the non-overlapping train and test data that include only one observation for each participant (Table A2). The second data included the most recent observation when two or more observations of a participant were selected for the train or test set. The robustness check results showed a comparable degree of classification performance across the algorithms and samples. Although this analysis does not conclusively rule out the potential overfitting of the model, it dissuades the concern that our main results were driven by the benefits of testing on more recent data of the participants who were already reflected in model training.

Table 4 presents the five most important predictors of suicide outcomes. The most predictive variables for suicidal ideation were mental health (CESD score and self-esteem), objective economic condition (income, consumption, net worth, and unpaid rent), satisfaction with family relations, life satisfaction, and smoking. For suicide planning or attempt, the most predictive variables were mental health (CESD score and self-esteem), objective economic condition (income and consumption), life satisfaction, smoking, and mother's educational background. The CESD score was consistently the top predictor of both suicidal outcomes, and the self-esteem score was the second most important predictor across the algorithms, except for the logistic regression of suicide planning or attempt.

**Table 4:** Top five most important predictors.

Algorithm:	Logistic	SVM	RF	XGBoost
Panel A: suicidal ideation (*N* = 3292)				
Feature 1	CESD score	CESD score	CESD score	CESD score
Feature 2	Self-esteem	Self-esteem	Self-esteem	Self-esteem
Feature 3	Satisfaction with family relation	Income	Income	Income
Feature 4	Smoking	Life satisfaction	Consumption	Satisfaction with family relation
Feature 5	Unpaid rent	Consumption	Net worth	Life satisfaction
Panel B: suicide planning or attempt (*N* = 488)				
Feature 1	CESD score	CESD score	CESD score	CESD score
Feature 2	Mother's education	Self-esteem	Self-esteem	Self-esteem
Feature 3	Smoking	Income	Income	Life satisfaction
Feature 4	Religion	Life satisfaction	Consumption	Income
Feature 5	Age	Consumption	Life satisfaction	Smoking

*Notes*: SVM, support vector machine; RF, random forest; XGBoost, extreme gradient boosting.

The same set of algorithms was trained with the CESD and self-esteem scores only (Table 5). Our goal here is to examine whether our algorithms can be developed into a more condensed version so that they can be used as a quick screening tool in primary care settings. The algorithms were tuned and cross-validated using the identical setup, and suicidal outcomes on the test set were predicted. The results showed that the algorithms with two predictors offered satisfactory classification performance for predicting suicidal ideation and suicide planning or attempt. These condensed algorithms showed slightly lower sensitivity and accuracy than the fully specified versions in Table 4, but still had sensitivity rates above 0.80 and accuracy in the range of 0.841–0.884. These results show the potential to develop a quick screening tool using the CESD and self-esteem scores.

**Table 5:** Algorithm performance for the reduced model.

	Logistic	SVM	RF	XGBoost
Panel A: suicidal ideation (*N* = 3292)				
Area under the curve	0.835	0.838	0.845	0.843
Sensitivity	0.801	0.804	0.839	0.829
Specificity	0.869	0.872	0.850	0.857
Positive predictive value	0.809	0.813	0.795	0.801
Negative predictive value	0.863	0.865	0.884	0.878
Accuracy	0.841	0.844	0.846	0.846
Panel B: suicide planning or attempt (*N* = 488)				
Area under the curve	0.869	0.877	0.865	0.869
Sensitivity	0.837	0.837	0.814	0.837
Specificity	0.900	0.917	0.917	0.900
Positive predictive value	0.857	0.878	0.875	0.857
Negative predictive value	0.885	0.887	0.873	0.885
Accuracy	0.874	0.884	0.874	0.874

*Notes*: SVM, support vector machine; RF, random forest; XGBoost, extreme gradient boosting.

## 4 Discussion

One of the challenges in suicide prevention is identifying a population subgroup that needs to be targeted by suicide-prevention campaigns and policies. Effective intervention requires credible information on which population subgroups are at greater risk and what makes them consider suicide. While there is extensive research on potential risk factors, relatively little research has linked this knowledge base to the *ex-ante* prediction of suicidal outcomes. In this study, we leveraged the ML approach and observational data of Korean adults to develop ML algorithms for screening individuals at risk for suicidal ideation and suicide planning or attempt. The potential risk factors were identified based on previous research and theory, and four ML algorithms were used to train the model. Our sample was obtained from the population survey data in which most responses were “stated” by a participant, as in primary care or community settings. Empirically, we used an undersampling method and feature selection to address the class imbalance problem and scalability issues.

All algorithms achieved satisfactory classification performance in the range of 80%–90% accuracy. XGBoost was the best performing model, with a sensitivity of 0.853 and accuracy of 0.863 for predicting suicidal ideation, and a sensitivity of 0.861 and accuracy of 0.884 for predicting suicide planning or attempt. Overall, the algorithms predicting suicide planning or attempt showed a slightly higher classification performance than the ones predicting suicidal ideation. We also found relatively small differences in classification performance across the linear and non-linear classifiers, as well as with the parametric and non-parametric algorithms. The most relevant predictors were depressive symptoms, self-esteem, and indicators of economic condition such as income, consumption, and net worth, in the order of predictive power. Finally, the algorithms including the top two predictors (depressive symptoms and self-esteem) showed a comparable degree of sensitivity and accuracy.

Our findings confirm the evidence that depressive symptoms and negative self-perception are salient risk factors for suicide (Bhar et al., 2008; Ducasse et al., 2018; Fredriksen et al., 2017; Raschke et al., 2022). Additionally uncovered in this study was that the ML algorithms could attain a satisfactory classification performance with a number of key mental traits of respondents. A plausible explanation is that depressive symptoms and self-esteem already explained a large fraction of variations in suicidal outcomes, and hence the extra risk factors contributed little predictive power to the algorithms. Psychological symptoms might be the downstream consequences of more fundamental conditions leading to suicide, which represents an opportunity to develop an effective screening tool. If such symptoms can be diagnosed with a short diagnostic instrument, one can develop a simple design logic that receives diagnostic inputs via online platforms or smartphone applications and convert them to the likelihood of committing suicidal acts. Those in the clinical setting may leverage this screening tool to identify suicide-prone individuals and offer them an appropriate counseling service or treatment in a timely manner.

The modeling results showed little difference in classification performance across the four ML algorithms. Several reasons may underlie this finding (Hand, 2006), but one might be related to the linear separability of our data. ML training for classification problems is a sequential process for determining the optimal decision boundary whereby data can be separated into two groups of outcome values. For data with a linearly separable pattern, a complex nonlinear classifier does not come with noticeable improvements in the classification performance but only takes more computational resources. Suicide prediction is a real-time task that requires rapid classification with reasonable accuracy. This goal would be best achieved by a simple multivariate approach such as logistic regression. Using a regression-based approach would allow quick assessment of suicide risk and enable the system to efficiently update model parameters as additional data accumulates in the clinical setting. It also has the benefits of high interpretability, which could tell clinicians important predictive markers of suicide and their relative effect sizes. The application of ML to suicide prediction needs to consider the context in which the algorithm is used, instead of pursuing more complex and computationally intensive algorithms.

The prediction model developed here may contribute to various dimensions of the existing suicide-prevention framework. For instance, primary care providers desiring to enhance their prevention efforts may use our models to develop an early warning system for individuals who are not yet clinically suicidal. This system will help care providers to identify lay persons who will likely progress to suicidal ideation and take preventive measures before related symptoms manifest. Those who are screened as at risk might be referred for an appropriate follow-up, such as counseling services and professional care by mental health clinicians. Furthermore, our models to predict suicide planning and attempt can be used to enhance therapeutic values of the existing anti-suicidal treatments and care. The randomized clinical trial of depressed individuals showed that anti-suicidal drugs produce greater curative effects in patients with the most severe suicidal symptoms, including frequent ideation and attempts (Grunebaum et al., 2012). Our prediction model has the potential to identify a subset of patients for whom active anti-suicidal treatments and care produce significant clinical benefits.

This study contributes to the literature in several ways. First, we offered rigorous evaluation of whether the ML-based suicide prediction has the potential to be implemented in the clinical setting. By demonstrating satisfactory classification performance of the reduced model, we highlighted the potential of a quick screening tool based on self-reported data. Our approach improves upon the previously developed ML algorithms, which used social network data or web data and hence could not be used in the public health setting (Cash et al., 2013; Jashinsky et al., 2014). We also expand the literature by examining both suicidal ideation and suicide planning or attempt. A majority of the existing research used suicidal ideation as a proxy for suicidal outcomes (Hill et al., 2017; Ryu et al., 2018), and thus could not examine whether the ML algorithms could identify those at higher risk of suicide. In this study, using data on suicide planning or attempt we demonstrated that the ML model based on survey data could identify those more prone to suicidal behaviors. Finally, we used a representative community sample of Korean adults to predict suicidal ideation and suicide planning or attempt. The existing research on suicidal ideation using the KoWePS focused on a single aspect of suicide risk markers (Kim & You, 2019; Pak & Choung, 2020; Yoon et al., 2017) and could not integrate this body of knowledge to develop a prediction model. In this study, we constructed a multivariate prediction model adjusted for demographic and psychological risk factors demonstrated in the literature. The included risk factors incorporate key aspects of empirical evidence and the underlying theories (Baumeister, 1990; Shneidman, 1993).

This study has several limitations. First, our study sample comprised groups of heterogeneous individuals ranging from college students to retirees. Since the predictors of suicide may differ across the life cycle, our approach of using a single algorithm to model the general population may not capture the risk factors that are more important for population subgroups (see Cho et al., 2021; Hung et al., 2015; Jung et al., 2019). Future research needs to develop ML algorithms at a more granular scale that suits distinctive patterns of suicidal risk for the elderly or youth. Second, self-reported data on suicide may be subject to some degree of social desirability bias (Macalli et al., 2021). In Korea, speaking about suicide or mental health issues is often considered taboo (Park & Kim, 2016), possibly causing a person to be labeled socially unfit or potentially dangerous to others. Under these circumstances, those who were concerned about their social image might have tried to cover up their suicidal symptoms or provided incorrect responses during the survey. This reporting bias may have manifested as additional noise in suicidal outcomes, reducing the model's explanatory power and out-of-sample generalizability. Future research may consider using the Social Desirability Scale (Crowne & Marlowe, 1960) to control for this bias or use an alternative survey method that does not require interactions with an interviewer (e.g., artificial intelligence interview). Lastly, we were unable to establish causality in the relationship between the potential risk factors and suicidal outcomes. The primary challenge to ML is that the data pattern learned from data does not represent cause-and-effect between the predictor and outcome. While we have demonstrated that suicidal risk can be predicted with a few psychosocial measures, there is no guarantee that manipulating these variables will alter suicidal outcomes later. Future research needs to use the causal ML model to identify the causal attributes of suicide.

## 5 Conclusion

This study has shown the potential of ML algorithms in classifying individuals who may consider suicide or plan and attempt suicide, in a population sample of community adults in Korea. Our findings contribute to the ongoing pursuit of fruitfully combining existing evidence with data science approaches, aiming to improve prospective identification of suicide-prone individuals. A relatively novel finding was that the ML algorithms taking only two predictors (depressive symptoms and self-esteem) showed comparable classification performance as the complex and resource-intensive ML algorithms. This study has important implications for both healthcare policy and clinical practice, given that a short diagnostic instrument based on self-reported data might allow early identification of individuals at high risk of suicidal ideation, and direct targeted interventions to those most likely to plan or attempt suicide.

## Ethics approval

This research received Institutional Review Board (IRB) exemption from the Bioethics Committee at the Sungkyunkwan University (no. 2022-07-037). Participants gave informed consent to participate in the study before taking part. The data were anonymized and linked by the Korea Institute for Health and Social Affairs before granting access for research.

## Funding

This research was supported by the BK21 FOUR (Fostering Outstanding Universities for Research) funded by the 10.13039/501100002701Ministry of Education (MOE) of Korea and 10.13039/501100003725National Research Foundation (NRF) of Korea.

## Author statement

**Jeongyoon Lee**: Conceptualization, Software, Formal analysis, Data curation, Writing - original draft. **Tae-Young Pak**: Conceptualization, Methodology, Software, Validation, Formal analysis, Writing - review & editing, Supervision, Funding acquisition.

## Patient consent for publication

Not required.

## Declaration of competing interest

None declared.

## Figures and Tables

**Table A1:** Algorithm performance, train and test set randomly drawn from the full sample.

	Logistic	SVM	RF	XGBoost
Panel A: suicidal ideation (*N* = 3292)				
Area under the curve	0.829	0.837	0.832	0.830
Sensitivity	0.797	0.806	0.819	0.815
Specificity	0.860	0.867	0.844	0.846
Positive predictive value	0.835	0.844	0.824	0.825
Negative predictive value	0.826	0.834	0.839	0.837
Accuracy	0.830	0.838	0.832	0.831
Panel B: suicide planning or attempt (*N* = 488)				
Area under the curve	0.852	0.843	0.869	0.869
Sensitivity	0.789	0.788	0.824	0.824
Specificity	0.915	0.898	0.915	0.915
Positive predictive value	0.931	0.918	0.933	0.933
Negative predictive value	0.750	0.747	0.783	0.783
Accuracy	0.840	0.833	0.861	0.861

*Notes*: SVM, support vector machine; RF, random forest; XGBoost, extreme gradient boosting.

**Table A2:** Algorithm performance, using one observation for each participant.

	Logistic	SVM	RF	XGBoost
Panel A: suicidal ideation (*N* = 2515)				
Area under the curve	0.827	0.836	0.847	0.838
Sensitivity	0.814	0.822	0.814	0.838
Specificity	0.861	0.871	0.881	0.878
Positive predictive value	0.803	0.817	0.810	0.804
Negative predictive value	0.861	0.867	0.883	0.874
Accuracy	0.839	0.849	0.855	0.847
Panel B: suicide planning or attempt (*N* = 438)				
Area under the curve	0.842	0.842	0.858	0.842
Sensitivity	0.800	0.800	0.800	0.800
Specificity	0.883	0.883	0.917	0.883
Positive predictive value	0.821	0.821	0.865	0.821
Negative predictive value	0.869	0.869	0.873	0.869
Accuracy	0.850	0.850	0.870	0.850

*Notes*: SVM, support vector machine; RF, random forest; XGBoost, extreme gradient boosting.

## Data Availability

The authors do not have permission to share data.

## Footnotes

1 The upper bound of 65 was determined by the cutoff age for Basic Pension in South Korea, a cash transfer program for older adults. Limiting the sample to respondents below age 65 helps minimize the potential confounding effects of government cash transfer on mental health and well-being outcomes (Pak, 2020, 2021).

2 An alternative technique to deal with imbalanced data is to classify target labels according to the optimal cutoff where sensitivity and specificity are jointly maximized (Xue et al., 2018). Our preliminary analyses confirmed that this alternative approach leads to comparable classification performance with the unseen data.
