# Supplementary material for: Machine learning prediction of suicidal ideation, planning, and attempt among Korean adults: A population-based study
Source: SSM Popul Health. 2022 Sep 14;19:101231. doi: 10.1016/j.ssmph.2022.101231 (PMC9573904; doi:10.1016/j.ssmph.2022.101231)
Supplement: Multimedia component 1 [file mmc1.pdf]

Appendix A. Supplementary data

Supplementary Table S1. Predictors selected for algorithm training

	Variables	Effect	Studies
Demographic characteristics	Age	(+)	Nock et al. (2008), Park and Kim (2016)
	Gender (1=female; 0=male)	(+)	Jeon et al. (2013), Nock et al. (2008)
	Education background (1=college or higher; 0=less)	(–)	Nock et al. (2008), Suh et al. (2021)
	Marital status (1=married; 0=never married, separated, divorced, or widowed)	(–)	Kim and Yoon (2018), Nock et al. (2008)
	No. of household members	(ind.)	Kong et al. (2022), Mittendorfer-Rutz et al. (2004)
	Employment status (1=working; 0=not working)	(–)	Yoon et al. (2017)
	Region of residence (1=Seoul, Incheon, Gyeonggi; 0=others)	(ind.)	Kong et al. (2022), You et al. (2011)
	Religion (1=any religion; 0=no)	(ind.)	Lawrence et al. (2016)
Socioeconomic status	Income (household income over 12 mo.)	(–)	Davison et al. (2015), Suh et al. (2021)
	Consumption (average monthly household consumption last year)	(–)	
	Net worth (household assets - total debt)	(–)	
	Social welfare receipt (1=any social welfare benefits; 0=none)	(+)	Choi et al. (2017)
	Skipped meal (1=skipped meal due to financial strain; 0=others)	(+)	Choi et al. (2021), Davison et al. (2015), Pryor et al. (2016)
	Marginal food security (1=unable to buy food due to financial strain; 0=others)	(+)	
	Low food security (1=unable to eat balanced meal due to financial strain; 0=others)	(+)	
	Very low food security (1=reduced or skipped meal due to financial strain; 0=others)	(+)	
	Limited food intake (1=eaten less than desired due to financial strain; 0=others)	(+)	
	Limited food access (1=could not eat when hungry due to financial strain; 0=others)	(+)	
	Unpaid rent (1=involuntary moving due to unpaid rent; 0=others)	(+)	Choi et al. (2021), Kim and You (2019)
	Unpaid utility bills (1=could not pay bills on time; 0=others)	(+)	
	Unpaid taxes (1=electricity, phone, water cut off due to unpaid taxes; 0=others)	(+)	
	Unpaid education fees (1=unpaid education expenses due to financial strain; 0=others)	(+)	
	Limited heating in winter (1= limited heating due to financial strain; 0=others)	(+)	
	Limited healthcare access (1=limited healthcare access due to financial strain; 0=others)	(+)	Choi et al. (2021), Kim and You (2019)
	Limited health insurance ownership (1=health insurance eligibility suspended due to non-payment; 0=others)	(+)	
	No. of health insurance coverages	(ind.)	
	No. of outpatient visits	(+)	
	Credit delinquency (1=any family member credit delinquent; 0=others)	(+)	Choi et al. (2021)
Health and well-being	Poor self-rated health (1=poor or very poor; 0=good or better)	(+)	Kim and Yoon (2018), Kim and You (2019)
	Disability (1=disabled; 0=not disabled)	(+)	
	Chronic disease (1=one or more chronic conditions;	(+)	

	0=none)		
	Smoking (1=currently smoking; 0=none)	(+)	Borges et al. (2006), Choi et al. (2017), Jeon et al. (2013)
	Drinking (1=currently drinking; 0=none)	(+)	
	Depression (CESD scale)	(+)	Bhar et al. (2008), Borges et al. (2006), Choi et al. (2021)
	Self-esteem (Rosenberg's self-esteem scale)	(–)	
	Satisfaction with family economic condition (1=satisfied; 0=not satisfied)	(–)	Heisel and Flett (2004), Park and Kim (2016), Suh et al. (2021)
	Satisfaction with living environment (1=satisfied; 0=not satisfied)	(–)	
	Satisfaction with family relation (1=satisfied; 0=not satisfied)	(–)	
	Satisfaction with social relation (1=satisfied; 0=not satisfied)	(–)	
	Job satisfaction (1=satisfied; 0=not satisfied)	(–)	
	Leisure satisfaction (1=satisfied; 0=not satisfied)	(–)	
	Life satisfaction (1=satisfied; 0=not satisfied)	(–)	
	Physical abuse by spouse (1=physically abused by spouse; 0=none)	(+)	Naved and Akhtar (2008)
Early life conditions	Region of residence in childhood (1=metropolitan areas; 0=other areas)	(ind.)	Jeon et al. (2013), Maynard et al. (2015), Stansfeld et al. (2017)
	Economic condition in childhood (1=poor; 0=not poor)	(–)	
	Dropping from school due to financial strain (1=yes; 0=no)	(+)	
	Grew up in relative's house due to financial strain (1=yes; 0=no)	(+)	
	Loss of parents in childhood (1=yes; 0=no)	(+)	
	Divorce of parents in childhood (1=yes; 0=no)	(+)	
	Father’s education background (1=high school or above; 0=less than high school)	(–)	Kim et al. (2019), Mittendorfer-Rutz et al. (2004)
	Mother’s education background (1=high school or above; 0=less than high school)	(–)	
	Father’s occupation (1=white collar; 0=others)	(ind.)	
	Mother's occupation (1=white collar; 0=others)	(ind.)	
	Inheritance from parents (1=yes; 0=no)	(ind.)	Ullah et al. (2021)
	Migration into South Korea (1=yes; 0=no)	(ind.)	Bahk et al. (2017)

Supplementary Table S2. Recursive feature elimination

Suicidal ideation (*N*=3292)	Suicide planning or attempt (*N*=488)
Age	Age
Gender	Gender
Education background	Education background
Marital status	Marital status
No. of household members	No. of household members
Employment status	Employment status
Region of residence	Region of residence
Religion	Religion
Income	Income
Consumption	Consumption
Net worth	Net worth
Social welfare receipt	Social welfare receipt
Skipped meal	Skipped meal
Marginal food security	Marginal food security
Low food security	Low food security
Very low food security	Very low food security
Limited food intake	Limited food intake
Limited food access	Limited food access
Unpaid rent	Unpaid rent
Unpaid utility bills	Unpaid utility bills
Unpaid taxes	Unpaid taxes
Unpaid education fees	Unpaid education fees
Limited heating in winter	Limited heating in winter
Limited healthcare access	Limited healthcare access
Limited health insurance ownership	Limited health insurance ownership
No. of health insurance coverages	No. of health insurance coverages
No. of outpatient visits	No. of outpatient visits
Credit delinquency	Credit delinquency
Poor self-rated health	Poor self-rated health
Disability	Disability
Any chronic disease	Any chronic disease
Smoking	Smoking
Drinking	Drinking
CESD score	CESD score
Self-esteem score	Self-esteem score
Satisfaction with family economic condition	Satisfaction with family economic condition
Satisfaction with living environment	Satisfaction with living environment
Satisfaction with family relation	Satisfaction with family relation
Satisfaction with social relation	Satisfaction with social relation
Job satisfaction	Job satisfaction
Leisure satisfaction	Leisure satisfaction
Life satisfaction	Life satisfaction
Physical abuse by spouse	Physical abuse by spouse
Region of residence in childhood	Region of residence in childhood
Economic condition in childhood	Economic condition in childhood
Dropping from school due to financial strain	Dropping from school due to financial strain
Grew up in relative's house due to financial strain	Grew up in relative's house due to financial strain
Loss of parents in childhood	Loss of parents in childhood
Divorce of parents in childhood	Divorce of parents in childhood
Father's education background	Father's education background
Mother's education background	Mother's education background
Father's occupation	Father's occupation in childhood
Mother's occupation	Mother's occupation in childhood
Inheritance from parents	Inheritance from parents
Migration into South Korea	Migration into South Korea

Note: the shaded area represents a predictor selected by recursive feature elimination.

References

Bahk, J., Kim, A. M., & Khang, Y. H. (2017). Associations of multicultural status with depressive mood and suicidality among Korean adolescents: The roles of parental country of birth and socioeconomic position. *BMC Public Health, 17*(1), 1–12.

Bhar, S., Ghahramanlou-Holloway, M., Brown, G., & Beck, A. T. (2008). Self-esteem and suicide ideation in psychiatric outpatients. *Suicide and Life-Threatening Behavior, 38*(5), 511–516.

Borges, G., Angst, J., Nock, M. K., Ruscio, A. M., Walters, E. E., & Kessler, R. C. (2006). A risk index for 12-month suicide attempts in the National Comorbidity Survey Replication (NCS-R). *Psychological Medicine, 36*(12), 1747–1757.

Choi, S. B., Lee, W., Yoon, J. H., Won, J. U., & Kim, D. W. (2017). Risk factors of suicide attempt among people with suicidal ideation in South Korea: A cross-sectional study. *BMC Public Health, 17*(1), 1–11.

Choi, M., Lim, J., Chang, S. S., Hwang, M., Kim, C. S., & Ki, M. (2021). Financial hardship and suicide ideation: Age and gender difference in a Korean panel study. *Journal of Affective Disorders, 294*, 889–896.

Davison, K. M., Marshall-Fabien, G. L., & Tecson, A. (2015). Association of moderate and severe food insecurity with suicidal ideation in adults: National survey data from three Canadian provinces. *Social Psychiatry and Psychiatric Epidemiology, 50*(6), 963–972.

Heisel, M. J., & Flett, G. L. (2004). Purpose in life, satisfaction with life, and suicide ideation in a clinical sample. *Journal of Psychopathology and Behavioral Assessment, 26*(2), 127–135.

Jeon, H. J., Hong, J. P., Fava, M., Mischoulon, D., Nyer, M., Inamori, A., … Cho, M. J. (2013). Childhood parental death and lifetime suicide attempt of the opposite gender offspring in a nationwide community sample of Korea. *Suicide and Life-Threatening Behavior, 43*(6), 598–610.

Kim, S. H., Kim, J. S., Yoo, H. Y., & Ryu, E. (2019). Parental occupational status and suicidal ideation in adolescent: Cross-sectional secondary data analysis. *Journal of Pediatric Nursing, 45*, e57–e63.

Kim, J., & Yoon, S. Y. (2018). Association between socioeconomic attainments and suicidal ideation by age groups in Korea. *International Journal of Social Psychiatry, 64*(7), 628–636.

Kim, S., & You, M. (2019). An empirical analysis of delayed monthly bill payments as an early risk factor of increased suicidal behavior. *International Journal of Environmental Research and Public Health, 16*(16), 2929.

Kong, K. A., Kim, Y. E., Lim, S., Kim, B. Y., Kim, G. E., & Kim, S. I. (2022). Depressive symptoms and suicidal ideation in individuals living alone in South Korea. *Diagnostics, 12*(3), 603.

Lawrence, R. E., Brent, D., Mann, J. J., Burke, A. K., Grunebaum, M. F., Galfalvy, H. C., & Oquendo, M. A. (2016). Religion as a risk factor for suicide attempt and suicide ideation among depressed patients. *The Journal of Nervous and Mental Disease, 204*(11), 845–850.

Maynard, B. R., Salas-Wright, C. P., & Vaughn, M. G. (2015). High school dropouts in emerging adulthood: Substance use, mental health problems, and crime. *Community Mental Health Journal, 51*(3), 289–299.

Mittendorfer-Rutz, E., Rasmussen, F., & Wasserman, D. (2004). Restricted fetal growth and adverse maternal psychosocial and socioeconomic conditions as risk factors for suicidal behaviour of offspring: A cohort study. *The Lancet, 364*(9440), 1135–1140.

Naved, R. T., & Akhtar, N. (2008). Spousal violence against women and suicidal ideation in Bangladesh. *Women's Health Issues, 18*(6), 442–452.

Nock, M. K., Borges, G., Bromet, E. J., Alonso, J., Angermeyer, M., Beautrais, A., … Williams, D. (2008). Cross-national prevalence and risk factors for suicidal ideation, plans and attempts. *The British Journal of Psychiatry, 192*(2), 98–105.

Park, E. Y., & Kim, J. H. (2016). Factors related to suicidal ideation in stroke patients in South Korea. *Journal of Mental Health, 25*(2), 109–113.

Pryor, L., Lioret, S., Van Der Waerden, J., Fombonne, ́E., Falissard, B., & Melchior, M. (2016). Food insecurity and mental health problems among a community sample of young adults. *Social Psychiatry and Psychiatric Epidemiology, 51*(8), 1073–1081.

Stansfeld, S. A., Clark, C., Smuk, M., Power, C., Davidson, T., & Rodgers, B. (2017). Childhood adversity and midlife suicidal ideation. *Psychological Medicine, 47*(2), 327–340.

Suh, W. Y., Lee, J., Yun, J. Y., Sim, J. A., & Yun, Y. H. (2021). A network analysis of suicidal ideation,

depressive symptoms, and subjective well-being in a community population. *Journal of Psychiatric Research, 142*, 263–271.

Ullah, Z., Shah, N. A., Khan, S. S., Ahmad, N., & Scholz, M. (2021). Mapping institutional interventions to mitigate suicides: A study of causes and prevention. *International Journal of Environmental Research and Public Health, 18*(20), 10880.

Yoon, S., Kim, J. Y., Park, J., & Kim, S. S. (2017). Loss of permanent employment and its association with suicidal ideation: A cohort study in South Korea. *Scandinavian Journal of Work, Environment & Health, 43*(5), 457–464.

You, S., Van Orden, K. A., & Conner, K. R. (2011). Social connections and suicidal thoughts and behavior. *Psychology of Addictive Behaviors, 25*(1), 180–184.
